# Leukocytoclastic Vasculitis: A Case Report

**DOI:** 10.7759/cureus.41736

**Published:** 2023-07-11

**Authors:** Mona J Malik, Muhammad Nabeel Pasha, Victor Salib

**Affiliations:** 1 Internal Medicine, Univeristy of California, Riverside, Riverside, USA; 2 Pulmonary and Critical Care Medicine, One Brooklyn Health, New York, USA; 3 Family Medicine, Univeristy of California, Riverside, Riverside, USA

**Keywords:** skin necrosis, iga-mediated immune complex, dermal hypersensitivity, raynaud's phenomenon, avascular necrosis, leukocytocsis vasculitis, neutrophils, fibrinoid, systemic

## Abstract

Leukocytoclastic vasculitis, also known as hypersensitivity angiitis, is a cutaneous, small vessel vasculitis of the dermal capillaries and venules. The predominant clinical presentation is palpable purpura. Multiple medications can cause leukocytoclastic vasculitis, as well as autoimmune diseases, infections, and malignancy. The disease process may be limited to only the skin or a manifestation of a systemic vasculitis or process. Treatment is centered on symptom management. Our patient is a 60-year-old female who presented with bilateral dry and wet tender ulcerations. She was previously treated with paclizumab.

## Introduction

Leukocytoclastic vasculitis, also known as hypersensitivity angiitis, is a cutaneous, small vessel vasculitis of the dermal capillaries and venules, usually in the lower extremities [1]. The condition can be idiopathic or associated with malignancy, medications, autoimmune conditions, and infections. Symptoms can present as itchiness or pain, or patients can be asymptomatic [2]. With about 30-45 cases per one million, it is a disease often treated with rest, leg elevation, compression stockings, and antihistamines. In chronic cases, a four to six-week course of steroids may be given [2].

## Case presentation

A 60-year-old female with past medical history of Raynaud's disease and prior diagnosis of leukocytoclastic vasculitis in 1999, presented with painful lower extremities for the past one week. Her legs had been wrapped with dressing in setting of chronic history of ulcerations and itching. The dressing was removed about a week ago and since then she had progressive worsening of pain and itching extending up to her thighs at the time of presentation. She had prior vasculitis flare-ups in 2001 and 2010. Her rheumatologist had recommended paclizumab per the patient but she refused to take any medication treatment due to fear of side affects. Her last appointment with her rheumatologist was five years ago. The patient was able to ambulate without help and was active. 

**Figure 1:**
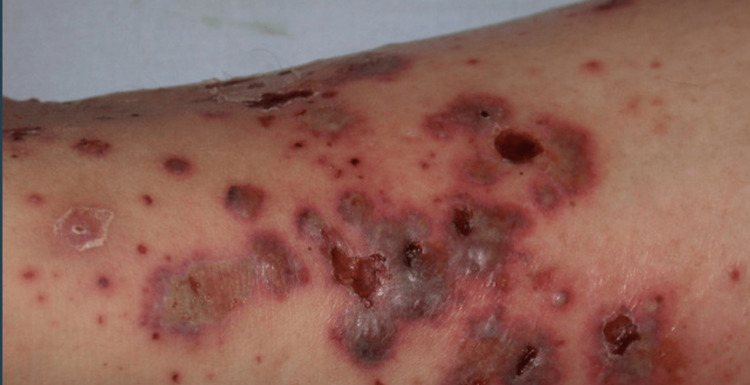
Bilateral vasculitis skin changes in the lower legs.

The patient denied symptoms of Raynaud's, including shortness of breath, difficulty swallowing, heartburn, and paraesthesias in fingers and toes. She does admit to fatigue and hardening of the skin in her extremities. Her vitals were stable. On physical exam, her upper and lower extremities were significant for diffuse livedo reticularis, more severe on her lower extremities. Multiple ulcerations were also noted on both extremities ranging from 0.25cm to 0.5cm in size, consisting of wet and dry granulation tissue (Figure 1). No bleeding or scaling was noted. Her skin was warm and erythematous. Pedis and popliteal pulses were intact, as was motor strength. There was tenderness on palpation of her shins bilaterally. No lesions were noted on her face, chest, or back. 

## Discussion

As mentioned, leukocytoclastic vasculitis is a rare condition of the cutaneous small vessels of the dermal capillaries and venules, often associated with autoimmune conditions. Classic histopathologic findings on biopsy are characterized by leukocytoclasis, which is vascular damage caused by nuclear debris from infiltrating neutrophils [1]. Histopathology often shows dilated vessels, hemosiderin deposits, red cell extravasation, and perivascular lymphohistiocytic infiltration. Although vasculitis can occur at any age, certain vasculitides occur at the end of the age spectrum, with Kawasaki disease in young children and giant cell arteritis in older adults. Small vessel vasculitis includes three main types: granulomatosis with polyangiitis (GPA), microscopic polyangiitis (PMA), and microscopic polyangiitis (MPA) [2]. The combined incidence rate from 1996-2015 was 33 per million in the USA [3].

Our patient also had Raynaud’s, a condition where some areas of the body can feel numb or cold in settings of low temperature, anxiety, or stress. Raynaud's can be a sign of underlying autoimmune conditions [1]. Triggers for leukocytoclastic vasculitis include autoimmune conditions like lupus, scleroderma, and rheumatoid arthritis. The condition can also be a manifestation of IgA-mediated immune complexes. It may also be known as Henoch-Schonlein purpura and can often presents with palpable purpura on the lower extremities [1]. 

Our patient's vasculitis may have been triggered by an infection or her health condition with Raynaud’s. About 10% of leukocytoclastic vasculitis is triggered by medications including penicillins, fluoroquinolones, valproic acid, phenytoin, anti-tumor necrosis factor (TNF) agents, and hydralazine. Among malignancies, blood cancers are found to have a link with leukocytoclastic vasculitis; it may also be connected to some infections such as hepatitis B and C [1]. 

Leukocytoclastic vasculitis is an inflammatory process, not a pathological one. The differential diagnosis includes cryoglobulinemic vasculitis, drug reactions, and Henoch-Schonlein purpura. Other causes of immune-complex vasculitis include inflammatory bowel disease, systemic lupus erythematosus (SLE), paraneoplastic phenomena, and infections [2]. Diagnosis requires a combination of clinical findings and serologic, pathologic, and diagnostic imaging studies [3]. The Chapel Hill Consensus Conference on the Nomenclature of Systemic Vasculitides proposes names and definitions for the most common forms of vasculitis. The usual distribution of vessel involvement is large vessel, medium vessel, and small vessel vasculitis [1]. The skin is commonly involved and is often a diagnostic tool with biopsy performed for histology [3]. The purpose of diagnostic criteria is to determine the specific type and extent of systemic involvement of leukocytoclastic vasculitis and to identify an underlying cause [4]. Work‐up should include the history of drug intake and preceding infections, biopsy with immunofluorescence, differential blood count, urine analysis, and throat swabs. An unregulated drive of the immune system results in neutrophil infiltration in the small vessels [4]. The neutrophils undergo degeneration known as leukocytoclasis with nuclear dust or karyorrhexis. Eventually, fibrinoid necrosis is evident throughout the vasculature [2].

The condition presents acutely and self-resolves in about 90% of cases. Avoidance of processed food and grain-fed meat can help alleviate the inflammation. In addition, vitamin C and vitamin E are adjuncts for treatment. Both vitamins C and E reduce superoxide production by neutrophils, mitigating the inflammatory response to trigger factors. Lastly, appropriate stress management will help to keep the leukocytoclastic inflammatory flare-ups in check [2]. Corticosteroids may be part of the treatment plan when there are signs of incipient skin necrosis [4]. In chronic or relapsing leukocytoclastic vasculitis, colchicine is used as a first‐line, and dapsone as a second‐line therapy [4]. 

## Conclusions

The patient in this report had presented to urgent care. She was counseled on the importance of scheduling an appointment with her rheumatologist and being open to receiving treatment for her leukocytoclastic vasculitis, given that her extremities had such a severely mottled appearance and she was experiencing pain. In the urgent care, we provided supportive care and pain management medication as needed. The patient agreed to see her rheumatologist to begin treatment.
